# Micropapillary Variant of Urothelial Carcinoma

**DOI:** 10.1155/2011/217153

**Published:** 2011-10-05

**Authors:** Ghee Young Kwon, Jae Y. Ro

**Affiliations:** ^1^Department of Pathology, Samsung Medical Center, Sungkyunkwan University School of Medicine, Seoul 135-710, Republic of Korea; ^2^Department of Pathology, The Methodist Hospital, Weill Medical College of Cornell University, Houston, TX 77030, USA; ^3^Department of Pathology, National Cancer Center, Gyeonggi-do 410-769, Republic of Korea

## Abstract

Micropapillary carcinoma (MPC) of urinary tract is an uncommon variant of urothelial carcinoma with significant diagnostic and prognostic implications. Though MPC shows characteristic microscopic features, there exists interobserver variability and also it needs to be differentiated from the metastasis from other organs. The prognosis is generally poor, depending on the proportion of the micropapillary component in some reports. Early cystectomy in cases with only lamina propria invasion may be indicated according to recent studies. This review outlines the general features of this entity and briefly comments on the controversies and the recent development.

## 1. Introduction

Micropapillary carcinoma (MPC) of the urinary tract is a well-recognized variant of urothelial carcinoma (UC) characterized by distinct histologic features and aggressive clinical course.  Table 1 shows urothelial carcinoma and its variants including MPC. MPC is worthy of note for its implications regarding both diagnosis and clinical management. Despite increased awareness of the entity and recent development in the understanding into its pathogenesis, there still exist controversies concerning certain aspects of this rare tumor. This paper will outline the general features of this entity and briefly comment on the controversies and the recent development.

## 2. Incidence

At least 500 cases of MPC of the bladder have been reported as a special variant since its first description in 1994 by Amin et al. [1], and MPC is reported to comprise 0.6 to 8.2% of urothelial carcinoma, with later series reporting the higher end of the spectrum [2, 3]. The recent rise in incidence appears to reflect the increased awareness of this entity, and the variable proportion occupied by this tumor is evidently due to a lack of established criteria for diagnosis and less-than-perfect interobserver reproducibility, both of which issue will be addressed later in this paper. This tumor predominantly affects male with male to female ratio of 5 : 1 to 10 : 1 which is higher than that for conventional UC which is 3 : 1 [1, 4–7].

## 3. Gross Features

Gross morphology of MPC is variable, and there are no unique features to distinguish it from conventional UC or other variants. MPC can present as papillary, sessile, polypoid, ulcerative, or infiltrative mass, and the size can also be variable from microscopic focus to over 10 cm [7].

## 4. Microscopic Features

**Figure 1:**
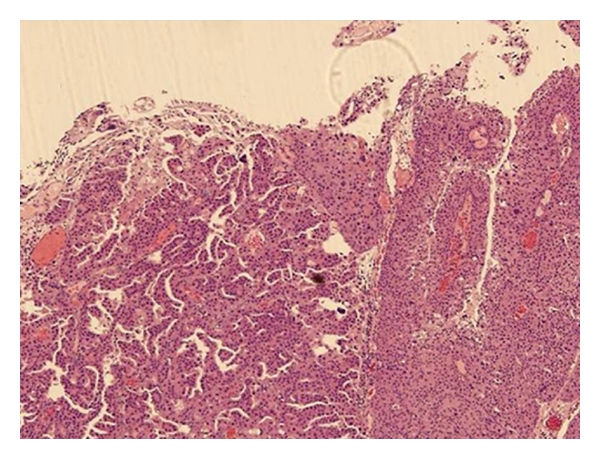
The MPC (left) shows delicate filiform projections on the mucosal surface, different from the ordinary type of papillary carcinoma (right).

**Figure 2:**
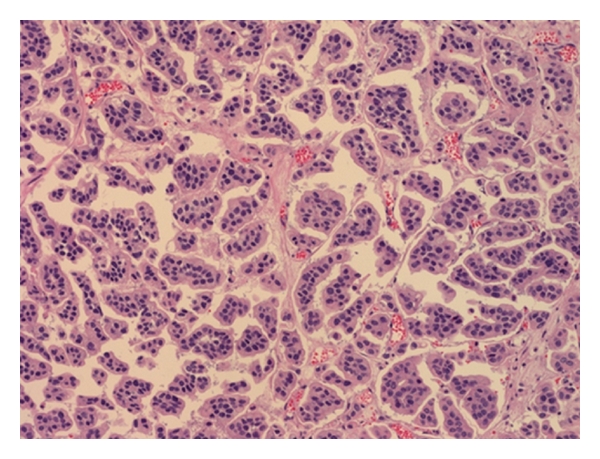
Deep invasive component of MPC displays tight nests or balls of tumor cells in lacuna-like spaces.

**Figure 3:**
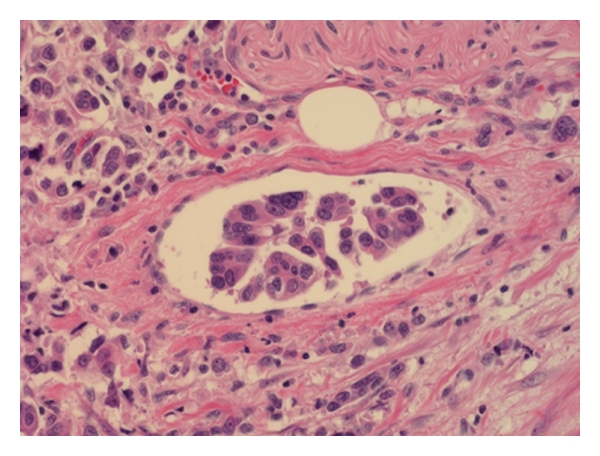
The tumor cells show high nuclear grade and reversed polarity.

**Figure 4:**
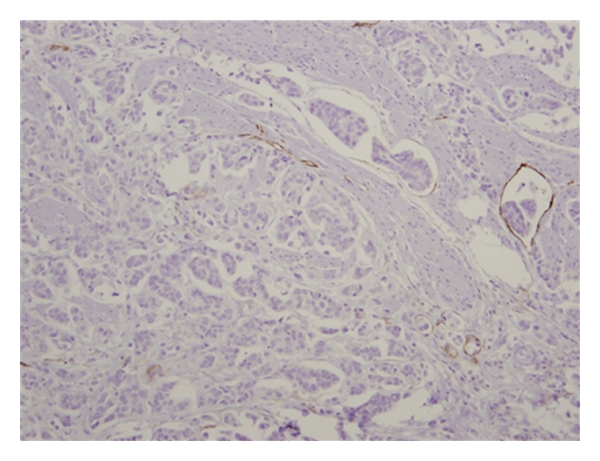
Immunostaining for D2-40 demonstrates a focus of lymphatic invasion. This stain proves that the bulk of clear lacunar spaces are not true lymphovascular spaces.

**Figure 5:**
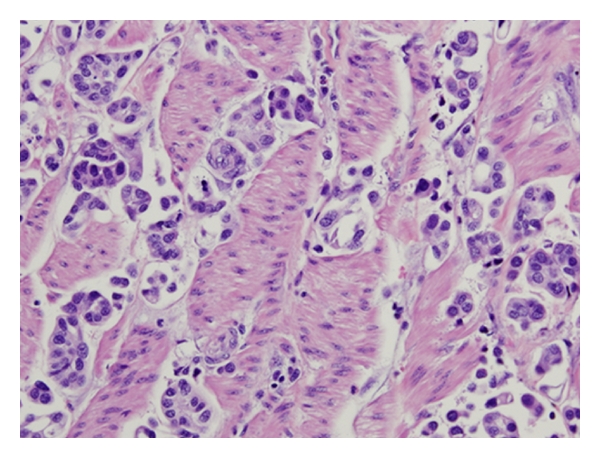
The tumor infiltrates between thick bundles of proper muscle layer.

The defining microscopic feature of MPC is micropapillary architecture reminiscent of the papillary configuration seen in ovarian papillary serous tumors. The micropapillary pattern of MPC can present either (i) on the mucosal surface as slender delicate processes which are usually devoid of a fibrovascular core and appear as glomeruloid bodies on cross section (Figure 1), or (ii) in the invasive component as small tight cell nests or balls contained in lacunae or stromal retraction spaces (Figure 2), mimicking lymphovascular invasion (LVI). The nuclei of tumor cells are frequently of high grade, showing reversed polarity to the external surface of tumor nests (Figure 3). A small proportion of the tumor-containing spaces represents actual lymphovascular invasion as evidenced by immunostaining for endothelial markers such as factor VIIIR-Ag, *Ulex europaeus* agglutinin I lectin, CD 31, CD34, and D2-40 (Figure 4). Although LVI is present in most cases of invasive MPC if adequately sampled and diligently searched for, a vast majority of the tumor-containing lacunae lack endothelial lining and do not constitute true LVI. Psammoma bodies, found in ovarian papillary serous neoplasia, are vanishingly rare in urinary tract MPC. The overwhelming majority of this tumor shows deep muscle invasion (Figure 5), and thus, it is recommended to alert clinicians regarding the invasive potential of this tumor when the biopsy is obtained mainly from the superficial layer and proper muscle is not included in the biopsy [1, 8]. 

MPC is usually accompanied by conventional UC, the proportion of MPC ranging from focal to almost exclusive. There are no established criteria for the cutoff proportion of MP component to qualify as MPC and some authors suggest 5% or 10% as the lower limit, while others adopt a noncommittal approach and render diagnosis using terminology such as “UC, high grade, with micropapillary histology (40%)” [9]. It is reported that the presence of any amount of MPC portends a poor outcome [5] and that a larger proportion of MP component is associated with more dismal clinical outcome [10]. Therefore, whichever criteria one may apply for the definition of MPC, it is recommended to report the presence and the proportion of MP component in the pathology report. MPC is frequently accompanied by in situ carcinoma, and coexistence has been reported with adenocarcinoma [11, 12], small cell carcinoma [12], sarcomatoid carcinoma [13], pleomorphic giant cell carcinoma [14], lipoid variant [15], or plasmacytoid variant of UC [16]. 

On urine cytology, the smear shows papillary/spheroid clusters of tumor cells showing a high nuclear grade. Solitary tumor cells are infrequent and the background is relatively clear, reflecting the growth pattern of the tumor which is usually transmural rather than superficial spread [17].

## 5. Differential Diagnosis and Ancillary Diagnostic Tests

The most important differential diagnosis for urinary tract MPC is its distinction from conventional UC with prominent retraction artifacts, which issue has been elegantly addressed in a recent consensus study by Sangoi et al. [6]. In that study, the agreement among uropathologists for the diagnosis of MPC was only moderate and the authors provide a few diagnostically useful morphologic observations. In their opinion, which is shared by us, multiple or small tumor nests in lacunar spaces are important diagnostic clues, while large or branching nests with anastomoses and confluence argue against the diagnosis of MPC. In this context, it appears that a sizable proportion of disagreement is from cases which display an intimate mixture of tumor nests displaying variable-sized tumor nests in diverse configuration. We suggest that it would be reasonable to diagnose MPC only when there is at least one high-power-field area of pure classic MPC without readily identifiable contradicting features. There are no immunohistochemical markers to reliably differentiate MPC from conventional UC. Though it has been reported that MUC1, CA125, Her/neu, and KL-6 might be specific for MPC [18], these results were not supported by other studies [19]. 

The next critical issue is the differential diagnosis from MPC of other organs in a metastatic setting. For example, in female patients presenting with abdominal metastasis, the main differential diagnosis is with metastatic papillary serous carcinoma of the ovary or primary peritoneal serous carcinoma. Clinical and radiologic correlation is mandatory, but there are a few helpful histologic and immunohistochemical features, such as the presence of typical UC or immunoreactivity to CK 20, high molecular weight cytokeratin, thrombomodulin, and uroplakin III in MPC of the bladder. Although uncommon, there also exist clinical situations where it is necessary to rule out metastatic MPC from other organs such as the lung, breast, pancreas, colon, stomach, or salivary glands. In such cases, the best immunohistochemistry panel is combining uroplakin III and CK20 (bladder), CK20 and CDX2 (colon), TTF-1 (lung), ER and mammaglobin (breast), and WT-1 and PAX8 (ovary) which can differentiate between primary cancers of urinary tract, lung, breast, ovary, and colon though pancreas and salivary gland are left with no specific markers [20].

## 6. Pathogenesis and Molecular Changes of MPC

There is a paucity of data regarding the pathogenesis of MPC. However, one interesting aspect is the reversed polarity of tumor cells. With that, tumor cells facing the stroma acquire apical secretory properties evidenced by ultrastructural examination and immunostaining for MUC1, a surface glycoprotein present on the apical/luminal surface. This unusual interface might lead to the detachment of tumor cells from the stroma, facilitating stromal invasion [21].

Some authors suggest that MPC may represent a form of glandular differentiation based on immunoreactivity to CA125 [10], and this appears to be a plausible argument, considering that MPC found in other organs is mostly a variant of adenocarcinoma. However, occasional expression of CA 125 in conventional UC, usual coexistence with conventional UC and rare expression of other markers of glandular differentiation such as MUC5A, MUC6, and CDX2 suggests that MPC of the bladder is of urothelial origin [22].

Another characteristic finding of MPC is the overexpression of Aurora A compared to conventional UC. Aurora A is a key player in maintaining genomic integrity, and its expression is associated with poor clinical outcome in bladder cancer. Enhanced expression of Aurora A may be a mechanism underlying increased chromosome copy number and total nuclear DNA content, contributing to the clinical aggressiveness of MPC [23].

On the level of molecular alterations, one early study has reported that p53 abnormalities are rare in MPC as compared to conventional UC, while the findings are the opposite for H-ras [24]. This implies distinct molecular pathways for MPC, but the number of cases was too small for generalization, and there has been no additional study to consolidate these findings.

## 7. Treatment and Clinical Course

Both clinical and pathological implication has undoubtedly contributed to the establishment of MPC as a distinct entity along with its wide recognition. Clinically, this tumor is almost invariably muscle invasive at the time of presentation with frequent metastasis to lymph nodes and distant organs. Thus, it is imperative to get a deep biopsy when the proper muscle invasion is not found on superficial sampling. 

Conventional paradigm for treatment of UC is implementing radical surgery in the muscle invasive diseases and intravesical BCG administration for the nonmuscle-invasive cases. While the conventional approach is applied in most institutions, early cystectomy for the nonmuscle-invasive MPC is advocated by one leading group on the ground that these tumors eventually develop muscle invasion and that the response to chemotherapy is limited when used as a secondary modality. Kamat et al. from MD Anderson Cancer Center reported a 10-year survival rate of 72% among patients who received early cystectomy for the nonmuscle-invasive disease, while none survived after the treatment according to the conventional paradigm [4]. Generally, clinical course is mostly poor with the 5-year and 10-year overall survival rates in the largest study being 74 and 54%, respectively [4]. 

Aggressive clinical behavior and the different treatment modality of MPC from other forms of UC illustrate the importance of correct recognition of MPC and the differential diagnosis of MPC from UC with tumor cells within artifactual tissue spaces, mimicking MPC.

## Figures and Tables

**Table 1:** Urothelial carcinoma and its variants.

Infiltrating urothelial carcinoma with squamous differentiation	
Infiltrating urothelial carcinoma with glandular differentiation	
Infiltrating urothelial carcinoma with trophoblastic differentiation	
Nested variant	
Microcystic variant	
Micropapillary variant	
Lymphoepithelioma-like variant	
Lymphoma-like and plasmacytoid variants	
Sarcomatoid variant	
Giant cell variant	
Undifferentiated	
